# The Community-Based Medication-First program for opioid use disorder: a hybrid implementation study protocol of a rapid access to buprenorphine program in Washington State

**DOI:** 10.1186/s13722-022-00315-4

**Published:** 2022-07-07

**Authors:** Caleb J. Banta-Green, Mandy D. Owens, Jason R. Williams, Jeanne M. Sears, Anthony S. Floyd, Wendy Williams-Gilbert, Susan Kingston

**Affiliations:** 1 Addictions, Drug & Alcohol Institute, University of Washington, Seattle, WA USA; 2 Department of Health Systems and Population Health, School of Public Health, University of Washington, Seattle, WA USA; 3 Harborview Injury Prevention and Research Center, University of Washington, Seattle, WA USA; 4 Institute for Work and Health, Toronto, ON Canada; 5 College of Nursing, Washington State University, Spokane, WA USA

**Keywords:** Medications for opioid use disorder, Opioid use disorder, Overdose, Implementation, Protocol

## Abstract

**Background:**

Opioid use disorder (OUD) is a serious health condition that is effectively treated with buprenorphine. However, only a minority of people with OUD are able to access buprenorphine. Many access points for buprenorphine have high barriers for initiation and retention. Health care and drug treatment systems have not been able to provide services to all—let alone the majority—who need it, and many with OUD report extreme challenges starting and staying on buprenorphine in those care settings. We describe the design and protocol for a study of a rapid access buprenorphine program model in six Washington State communities at existing sites serving people who are unhoused and/or using syringe services programs. This study aimed to test the effectiveness of a Community-Based Medication-First Program model.

**Methods:**

We are conducting a hybrid effectiveness-implementation study of a rapid access buprenorphine model of care staffed by prescribers, nurse care managers, and care navigators. The Community-Based Medication-First model of care was designed as a 6-month, induction-stabilization-transition model to be delivered between 2019 and 2022. Effectiveness outcomes will be tested by comparing the intervention group with a comparison group derived from state records of people who had OUD. Construction of the comparison group will align characteristics such as geography, demographics, historical rates of arrests, OUD medication, and health care utilization, using restriction and propensity score techniques. Outcomes will include arrests, emergency and inpatient health care utilization, and mortality rates. Descriptive statistics for buprenorphine utilization patterns during the intervention period will be documented with the prescription drug monitoring program.

**Discussion:**

Results of this study will help determine the effectiveness of the intervention. Given the serious population-level and individual-level impacts of OUD, it is essential that services be readily available to all people with OUD, including those who cannot readily access care due to their circumstances, capacity, preferences, and related systems barriers.

## Background

Expanding access to medications for opioid use disorder (MOUD) beyond traditional health care settings is essential given that few individuals with opioid use disorder (OUD) receive specialty addiction treatment (20%), and even fewer receive MOUD [1]. This gap persists despite the effectiveness of MOUD in reducing risk of overdose and death [2, 3]. These medications are underutilized in part because populations at elevated risk of death cannot easily access health care settings, appointment-based care and, in turn, medications. Other barriers may include: required abstinence from controlled substances and alcohol; complicating social, physical, and behavioral health conditions, such as chronic disease, lack of housing, incarceration, and mental illness; clinic-mandated counseling that patients find onerous, unhelpful, or aversive; and previous negative experiences with health care providers. Barriers to initiating and staying on MOUD have been described in terms of both accessibility and design [4]. Despite the large treatment gap, most people who use illicit opioids do not want to be using them and most do want to use treatment medications if they are easy to access [5, 6].

OUD is treatable, and the most effective treatments are the medications methadone and buprenorphine. Not only do they reduce mortality by approximately 50%, they support remission from active OUD, long-term recovery, and restoration of health and functioning across life domains [2, 3, 7–9]. Methadone faces regulatory barriers in the United States to ready access in many communities, primarily the need for near daily dispensing through opioid treatment programs. Buprenorphine has the advantage of being available via prescription from providers-waivered by the Drug Enforcement Administration (DEA) and obtained at pharmacies making it, potentially, widely available. Long-acting naltrexone, the third medication for OUD approved by the Food and Drug Administration (FDA) has been found in real-world studies to have modest uptake and retention, limiting the theoretical benefits of this medication [3, 7, 10].

Strategies that may help to increase providers’ uptake of buprenorphine prescribing include the nurse care manager model, whereby a nurse provides a substantial amount of care and interaction with patients, supporting prescribers to provide care for a larger number of patients on buprenorphine than they would otherwise [11–13]. Models that reduce barriers to care for patients are promising, and the rationale for these lower barrier models of care has been well articulated [14, 15]. Care navigation services are increasing nationally and show promise as a means to engage and retain clients in accessing health insurance and for engagement in various chronic disease models of care [16–20]. A national call for new models of care to improve access to MOUD has been articulated [21]. We have combined multiple aspects of promising models in a Community-Based Medication-First (CBMF) care model which we propose to test with the protocol described here to meet this need.

## Methods/design

### Objectives and hypotheses

We are conducting a pragmatic type 1 hybrid effectiveness-implementation study of a rapid access buprenorphine model of care, staffed by prescribers, nurse care managers, and care navigators in community-based settings. We are testing effects of the intervention on several outcomes while also observing and gathering information on the process of implementation [22].

The primary aim is to test the effectiveness of the intervention on morbidity and mortality outcomes. These outcomes are documented with Washington State data systems including arrest records, Medicaid claims for emergency and inpatient health care utilization, statewide hospitalization data, and the state death registry. We hypothesize lower rates for each of these measures for the intervention group than for a matched comparison group of people with OUD.

Secondary objectives of the study include: (1) evaluating whether sex, age, or baseline housing status modify the impact of the intervention; and (2) identifying barriers and facilitators to implementation.

### Implementation research overview

Consistent with a type-1 hybrid effectiveness-implementation study approach [22], we assessed the delivery of Meds First services and the potential for implementation of this model in addition to conducting the effectiveness trial. We collected basic information on monthly MOUD inductions and care navigation services. We also coordinated a co-design team of eight Meds First staff and conducted semi-structured qualitative interviews to inform the development of a Meds First Toolkit for implementation at future sites.

### Study design

A quasi-experimental study design is being used to test the impact of CBMF delivered to participants in six Washington State communities. A comparison group will be identified from state records of people with indicators of OUD. Construction of the comparison group will align characteristics such as geography, demographics, historical rates of arrests, MOUD history, and health care utilization, using restriction and propensity score techniques.

### Study timeline

Initial funding for the study was received on December 18, 2018. Human subjects’ materials development and review, data sharing agreement development, and site recruitment, selection, contracting, and training were carried out through June 30, 2019. The clinical intervention was implemented, and study recruitment and enrollment began on August 16, 2019. The last day of new clients beginning the clinical intervention was September 30, 2021, with up to 8 weeks for potential enrollment in the study. The intervention lasts 6 months, with study-funded clinical services ending March 31, 2022. A minimum of 12 months of follow-up data from the date of beginning the clinical intervention will be collected on all intervention participants; thus, data used for analyses will extend through September 30, 2022. Data analyses are expected to be completed by March 31, 2023.

### Intervention group participants

Participants are recruited from individuals presenting at a CBMF site for and receiving a buprenorphine prescription for an indication of OUD by a licensed health care provider authorized to prescribe buprenorphine for OUD (i.e., received a DATA 2000 waiver from the Drug Enforcement Administration). Potential participants must be between the ages of 18 and 70, willing to provide permission to access state records, and not already stabilized on MOUD. Stabilization is defined as receiving OUD medications for 15 or more days in the prior 30 days (excluding time in a controlled environment such as jail or hospital where they could not receive MOUD). Sufficient English language comprehension to understand the consent process is necessary for participation.

### Comparison condition

A comparison group will be drawn from WA Department of Social and Health Services (DSHS) Research and Data Analysis (RDA) records for people who have received publicly funded health care and have documentation of an OUD diagnosis. The comparison group data are being obtained per approval of a waiver of consent for WA State records data. Direct identifiers of those in the comparison group will not be provided to the study team. Some clients who participate in CBMF will not choose to enroll in or may be ineligible for the study. We do not have identifiers for these clients, so to maximize the chances that they are not inadvertently included in the comparison group, we will exclude from the comparison group all people who received MOUD from a CBMF-affiliated prescriber, even though they may not have received it via the CBMF program.

### The CBMF intervention

As described in our site recruitment materials “The Medication-First model of care is based upon rapid, typically same-day, access to medications; convenient, non-appointment based care; no exclusions for poly-substance use; no counseling mandates (but services readily available); and ongoing, easy to access care.”

Staffing for CBMF is based upon project-funded care teams comprised of a nurse care manager and care navigators, as well as a prescriber’s time to oversee clinical activities. Administrative and lease costs are also covered with project funds.

All clinics begin with drop-in, same-day visits (no appointment required) with work flows designed to provide same-day medication starts when medically appropriate and desired by clients. Brochures and posters for the CBMF project are placed in common spaces of the organizations. Staff of onsite partner agencies and programs [e.g., Syringe Services Programs (SSPs)] are educated about the CBMF project and able to provide referrals. CBMF staff are often co-located at SSPs to facilitate linkage. Due to SARS-CoV-2 infection concerns, some programs temporally instituted appointment-based care to minimize the number of clients in the clinic spaces beginning in March 2020.

Initial training and ongoing, twice-monthly and ad hoc technical assistance and clinical consultation are provided by our intervention support team for nurse care managers and care navigators. These calls address clinical and research issues. Separate support is provided to prescribers in monthly calls with the study team and focuses primarily on clinical issues. The intervention support team is composed of a registered nurse, a clinical psychologist, a social worker, and a physician with training in addiction medicine. Site administrators meet with the team twice monthly to discuss administrative, clinical, and research issues.

Recognizing that settings offering buprenorphine are mostly in health care systems with appointment-based care, often with many prerequisites, we sought to design and implement a model that actively reduces treatment barriers, many of which have been detailed previously [4, 24]. The program is intended for people who may have barriers to appointment-based care, including those who are unhoused. People accessing buprenorphine at SSPs are often more likely to be unhoused than those receiving care in outpatient medical settings. For example, in Seattle, Washington, 82% of Buprenorphine Pathways low barrier care participants reported current homelessness/unstable housing compared to 32% of participants in an office-based buprenorphine treatment program in an academic primary care clinic [25, 26].

Additionally, the intent of the model is to serve those who use multiple other drugs and alcohol, as they may be ineligible to start buprenorphine in many settings or may be discharged due to ongoing substance use. The Washington State SSP participant survey indicated that a majority of those who reported opioids as their main drug had also used methamphetamine in the prior three months (78%) [27]. Research has found that those who have OUD and use methamphetamine can be successfully treated for their OUD even while methamphetamine use continues. The Buprenorphine Pathways analysis found that a majority of buprenorphine clients also used methamphetamine at baseline, but that use was not associated with lower retention [26]. While methamphetamine was associated with lower retention in an academic primary care buprenorphine clinic overall, those who stayed on buprenorphine reduced their methamphetamine use [25]. Benzodiazepines are another commonly used substance among people with OUD, and some providers identify co-use of benzodiazepines as a reason to not begin or continue a person on buprenorphine. Of those reporting heroin as their primary drug surveyed at WA State SSPs in 2017, 34% reported recent benzodiazepine, tranquilizer or muscle relaxant use [27]. The FDA issued guidance in 2017 encouraging buprenorphine induction and maintenance for those using benzodiazepines or other central nervous system depressants whether prescribed to them or not, noting “The combined use of these drugs increases the risk of serious side effects; however, the harm caused by untreated opioid addiction can outweigh these risks” [28].

### Settings

Settings include existing program locations providing social and health services for unhoused people and SSPs. The goal of the purposeful site selection was to obtain geographic variability. Three of the sites are in Eastern Washington (Spokane, Walla Walla, Kennewick) and three in Western Washington (Tacoma, Seattle, Centralia). In Tacoma, the project is in a health department that is adjacent to a SSP run by a non-profit harm reduction organization. In Spokane, the initial site was in the county health department which also houses and operates an SSP. The Spokane site was moved to a community-based agency that provides day services for unhoused people and outpatient addiction treatment. The site in Walla Walla is in a not-for-profit, community-based HIV case management organization that also has an onsite SSP. This same organization also manages a SSP in Kennewick that also serves as a CBMF site. A small primary care clinic in Seattle is in a thrift store on a highway, with an adjacent mobile SSP; this clinic is affiliated with a local network of Federally Qualified Health Centers. A community-based organization operated by a church that also provides a clothing and food pantry, housing services, and an SSP, operates the program in Centralia.

The goal of this project is to provide services where people with OUD already receive other services and often have established trusting relationships with staff and volunteers. SSPs were selected as one of the settings for several reasons. Washington State SSP participant surveys have shown high interest in reducing opioid use and receiving buprenorphine or methadone in a 2015 survey [5]. The 2017 WA State SSP survey found that over half of respondents had felt they needed medical care at some point in the last year but did not seek it because of previous negative experiences with providers due to their substance use [27]. The 2015 survey findings motivated the Buprenorphine Pathways program in Seattle [26], the findings of which further motivated this study. Additionally, staff at the Addictions, Drug & Alcohol Institute (ADAI) had established relationships with many SSPs during the course of client surveying in 2015 as well as the implementation of a SAMHSA-funded overdose prevention and naloxone distribution program administered by staff at ADAI beginning in 2016. We also chose to work with other types of programs to explore whether and how the model worked at community-based settings, not solely SSPs. Adding other care setting types is important to add variability and help with generalizability. While many towns, tribes, cities, and entire states in the United States do not have SSPs, there are service providers providing other services to marginalized people with OUD.

### Study recruitment and enrollment procedures

After patients meet with medical staff and are informed they will be prescribed buprenorphine, they are asked about their interest in study participation by care navigators or the nurse care manager. These staff use a script to determine whether the person wants to know more about the study. If the person agrees, an informed consent process is conducted, and written consent and HIPAA authorization are obtained. These agency staff completed human subjects training and are documented research staff with the two relevant human subjects review agencies.

Research recruitment and enrollment can occur within eight weeks of the initial prescribing visit, whether or not clients obtain or use the buprenorphine. Because study participation only involves permission to access secondary data and there are no baseline interview data, his extra time does not negatively impact the completeness of data. Participants receive a $20 gift card at the time of approach for participation when they are told about the types and sources of data to be used. The research procedures involving identifiable information are a one-time request for administrative records from Washington State. Participants do not participate in any further research activity after the collection of identifiers used for administrative data linkage.

### Human subjects review

According to the Split Review Agreement with Washington State Institutional Review Board (IRB), the University of Washington IRB reviewed the recruitment, consent, and HIPAA authorization activities for the intervention group and all access to records that are not from the Washington State Department of Health or Health Care Authority. The University of Washington IRB determined that the application qualified for expedited review (“minimal risk”) and was approved on March 21, 2019 (IRB ID# STUDY00006623). The University of Washington IRB also determined that our study was not a clinical trial and it was not registered as one. The Washington State IRB reviewed the use of state-held secondary data records and approved the application under expedited review authority on June 25, 2019 (Project # 2019-032). Due to the use of identifiable records provided to RDA we completed confidentiality agreements with each state agency providing data.

### Data sources, linking, and sharing

Data collected at baseline include name, alternative name(s) and aliases, sex, date of birth, and Social Security number. In addition, we collect the date of first receiving a buprenorphine prescription in the CBMF program and self-reported housing status. These data will be provided to RDA to create a linked dataset of those receiving the intervention.

Data sources will include: DSHS Client Outcomes Database, which includes arrest records (from Washington State Patrol’s statewide database for all local law enforcement), along with records of hospitalizations, emergency department utilization and MOUD (i.e., methadone, buprenorphine, naltrexone) from Health Care Authority’s ProviderOne database. Comprehensive Hospital Abstract Reporting System hospitalization data and the Prescription Monitoring Program data for buprenorphine prescriptions (from WA Department of Health) will also be included. These data will be made available by the data holders to RDA, who will in turn create the linkage, build analytic datasets, remove links, and provide the limited, de-identified database for both intervention and comparison groups to the research team at ADAI.

Specific arrest data include the number and type of felony and gross misdemeanor charges per month. Hospitalization data elements include hospital admission types, dates of service, referral sources, discharge status, payers, and diagnosis and procedure codes. Health Care Authority ProviderOne data include medical diagnosis and procedure codes, prescription details and dates, and care settings. ProviderOne data also include dates of service in opioid treatment programs as well as prescribing data for naltrexone associated with OUD diagnoses so we can examine MOUD utilization. Prescription Monitoring Program data for all buprenorphine dispensing in Washington State will be provided with the complete prescription record including national drug code, quantity, days’ supply, dosage, date written, date filled, partial fill, and drug name as well as the National Provider Identifier. RDA also creates summary variables from ProviderOne including OUD diagnoses that will be used to help identify the comparison group.

Data sharing is guided by a funder requirement that raw project data be made “generally available to the public under the Open Source License for the duration of the grant period and for 3 years thereafter.” Secondary data including treatment and health care utilization and arrests will be stored. Study arm assignment will also be stored. These data are not stored at ADAI with links to identifiers.

### Outcomes

The primary effectiveness outcomes are derived from felony and gross misdemeanor arrests, Medicaid claims for emergency and inpatient health care utilization, statewide hospitalization data and the state death registry. For hospitalizations and emergency and inpatient care, we will examine any admissions and those associated with OUD or overdose. For deaths we will examine the overall death rate as well as drug poisonings involving opioids. ICD codes T40.0-T40.4 and T40.6 will be used to identify opioid poisonings among all drug poisonings (underlying cause of death in ICD X40-X44, X60-X64, X85, and Y10-Y14). Outcomes for the implementation research component include describing facilitators and barriers to the implementation of the intervention in each of the care settings for each of the care provider types.

### Analytic plan and power calculation

We plan an intent-to-treat analysis for all research-enrolled participants who received a prescription for buprenorphine, regardless of whether they actually obtained the medication. Those in the intervention group will be compared with those identified in the comparison group as having a history of OUD. The comparison group will be restricted to those who live in CBMF program counties or neighboring counties. Propensity score techniques will be used to align the intervention and comparison groups on demographics (age, gender); acute health care utilization (emergency department utilization and hospitalization), opioid treatment medication history (methadone, buprenorphine, and/or naltrexone); and arrest history.

We will construct the comparison group to create at least a 3:1 (comparison:intervention) match, without replacement. We anticipate utilizing negative binomial regression for comparing the resulting groups on count data such as emergency department utilization and survival analyses for time-to-event outcomes. Final modeling will be based upon data distributions and model fit. We will use robust standard errors, clustered by prescriber, to account for within-site correlation.

The target enrollment number is based upon an estimated annual non-fatal opioid overdose rate of 10% documented in emergency medical care records; the actual occurrence of non-fatal heroin overdose is approximately 20% [29] with approximately half seeking acute medical care during a serious overdose (unpublished data from the Washington State naloxone distribution project participant surveys managed by SK). Unfortunately, the fatal overdose rate locally and nationally has increased rapidly coinciding with increased fentanyl availability [30, 31], and overdose rates among those with a history of OUD may be much higher than when heroin was the predominant opioid [32]. The power calculation for detecting a difference in the proportions 10% vs 6.3% (37% reduction based upon Larochelle and colleagues [3]) results in 855 people in the intervention group for a confidence interval of 95% for a 2-sided test and 80% power to detect a significant difference.

For the implementation research components, there were 33 interviews, including group interviews with each of the six sites and individual interviews with 27 Meds First staff. Interviews were transcribed and will be analyzed using deductive and inductive thematic analysis supported by Dedoose qualitative analysis software. Additional content from the analysis of transcripts will support further development of the implementation toolkit and the use of peers in the CBMF model [23].

## Discussion

This protocol describes a pragmatic type 1 hybrid effectiveness-implementation trial that aims to test the CBMF model compared to usual care in the community using a quasi-experimental design. Given our previous experiences conducting randomized controlled trials and surveys with people with OUD, we determined a quasi-experimental study best fits the study goals, population of interest, model of care, and care settings. In our previous work, large proportions of participants, often the majority, were unhoused, dealing with histories of substantial trauma, and had multiple comorbidities. Given that the intervention was designed to be very low-barrier and rapid access, we felt it would be discordant to implement an RCT as it would be onerous on clinic staff and potentially interfere with clinical care. This meant balancing a less rigorous study design that would allow for causal inference with one that may yield higher recruitment rates and a study sample more likely representative of the overall intended clinic population.

The primary outcomes for this study will come from state administrative data, which was a study design element chosen to reduce or eliminate missing data. Attrition bias and variability in results after accounting for missing data are major concerns in the substance use treatment literature [33, 34]. The use of administrative data stands to reduce these biases and will allow for the examination of data for participants that could have been lost to follow-up, including those with the most severe OUD and those who disengage from treatment. The use of administrative data also reduces the burden on staff and participants that comes with extensive assessment batteries.

The pragmatic design and implementation in diverse settings enhance external validity and generalizability of findings. This study is being conducted at rural and urban sites and in different types of community-based organizations across Washington State, which will help to increase understanding of whether and how this program can be implemented in diverse locations. The implementation research elements of this study will be reported elsewhere.

While using administrative data is a significant strength of this study design because it reduces missing data and burden, it also comes with limitations. More extensive self-reported measures that could better describe the sample and capture more nuanced outcomes are not used. For example, it would be helpful to assess in-depth demographic characteristics, such as income and education; detailed measures of substance use e.g. type of opioid(s) or experiences in treatment. Illicitly manufactured fentanyl has emerged rapidly in the drug market in Washington State during the study period. Fentanyl dependence can complicate buprenorphine starts and increase overdose rates and therefore may impact our intervention and outcomes. We will discuss the impacts of this potential confounding in our outcomes paper [32, 35]. Additionally, there were no measures of fidelity for the CBMF program or systematic quantification of receipt of services by individual participant (e.g., to measure a dose–response effect). If this CBMF program shows promising outcomes, future studies should collect more self-report and clinical data to better understand how and for whom this program works and, potentially, does not.

## Conclusions

Existing gaps in receipt of MOUD and other treatments for OUD may be due in part to the numerous barriers individuals with OUD experience when seeking care in traditional healthcare settings, such as lack of prescribers in their community, lack of transportation, and costs [36, 37]. Individuals with OUD also experience stigma when presenting to hospitals and primary care [38, 39]. Stigma can make individuals less likely to seek care in the future [38]. Thus, providing person-centered care in trusted settings should help to expand access to and retention in MOUD [40], allow for opportunities to treat OUD sooner and more consistently, and consequently reduce opioid overdose across the United States.

## Data Availability

The datasets used and/or analyzed during the current study are available from the corresponding author on reasonable request.

## Abbreviations

ADAI: Addictions, Drug & Alcohol Institute
CBMF: Community based Medications First
DSHS: Department of Social and Health Services
DEA: Drug Enforcement Administration
FDA: Food and Drug Administration
HIPAA: Health Insurance Portability and Accountability Act
ICD: International Classification of Disease
IRB: Institutional Review Board
MOUD: Medications for opioid use disorder
OUD: Opioid use disorder
RDA: Research and data analysis
SAMHSA: Substance Abuse Mental Health Services Administration
SSP: Syringe Services Program
